# Potential effects of GPS collars on the behaviour of two red pandas (*Ailurus fulgens*) in Rotterdam Zoo

**DOI:** 10.1371/journal.pone.0252456

**Published:** 2021-06-04

**Authors:** Wiene van de Bunte, Janno Weerman, Anouschka R. Hof

**Affiliations:** 1 Wildlife Ecology and Conservation Group, Wageningen University, Wageningen, The Netherlands; 2 Royal Rotterdam Zoological & Botanical Gardens, Rotterdam, The Netherlands; 3 Department of Wildlife, Fish, and Environmental Studies, Swedish University of Agricultural Sciences, Umeå, Sweden; Feroze Gandhi Degree College, INDIA

## Abstract

GPS collars are frequently used to study the (behavioural) ecology of species. However, such collars can cause behavioural changes and can have negative physiological effects on the individuals wearing them. A pilot study to obtain data on behavioural and physiological effects of GPS collars on the target species would therefore be recommended, especially when it concerns rare or endangered species. The red panda (*Ailurus fulgens*) is a small carnivore endemic to the mountains of Central Asia that is currently classified as endangered. There is a lack in knowledge on the species ecology which could be enhanced by a study using GPS-technology. As a pilot study, the two adult red pandas in Rotterdam Zoo were observed before and after fitting a GPS-collar, to determine possible behavioural effects of wearing a collar. Although the study did not take place under ideal circumstances, indications of both behavioural, e.g. increased shaking behaviour, and physical, e.g. abrasions, effects of the collar were found. Even though our results were only based on two individuals, our findings stress the need for pilot studies in controlled environments before GPS collars to ensure safety of the study species and validity of the collected data.

## Introduction

Ecological research is one of the key drivers of species conservation. A relatively recent, powerful tool to obtain valuable data on species’ ecology is animal-borne GPS tracking [1, 2]. Through collaring, tagging or putting on a harness, a GPS transponder is fitted to the animal to follow its movements and e.g. study its behaviour. However, the question is if such devices alter the animals’ behaviour and if it will constrain the animal in any way, or even lower its survival. And, if possible changes in behaviour impact the results of the study. In the past it has generally been assumed that the fitting of a data transponder would have no effect on the animal and its behaviour [3]. Whereas this assumption may hold true for some species, it does not for others. Impacts of devices on the tagged individuals include decreases in activity and feeding [4, 5], alterations in movements [6], hair loss and skin abrasions [7, 8], and mortality rates [9, 10]. The impact of such devices on animals therefore needs to be tested for the sake of animal welfare as well as for the sake of the reliability of data collected when using transponders. This is especially important when it is the aim to collect data of sensitive species.

We studied the effects of GPS collars on the red panda (*Ailurus fulgens*), a small carnivorous mammal originating from the mountains of Central Asia. The red panda is currently listed as endangered and has a decreasing population trend [11]. To be able to conserve the species effectively, it is important to have a good knowledge of the species. The red panda has both been studied *in situ* and *ex situ*, but much knowledge is lacking on how red pandas interact with human-dominated landscapes. For instance, data on the temporal pattern of space use, resource use, movement, and social interaction, as well as the response to human disturbance, cattle grazing and habitat fragmentation by roads are lacking. Such data can be collected using tracking devices. So far, to our knowledge only four studies in the published literature, all in situ, have made use of tracking using collars [12–15]. All of these studies made use of radio telemetry and only one mentioned a possible effect of the radio collars. Yonzon and Hunter [14] found that direct observation of radio-collared red pandas was extremely difficult, because the animals showed aberrant behaviour immediately after seeing the investigators. What this behaviour entails specifically was not mentioned. The effects of tracking devices on the red panda therefore needs to be assessed to better assess the impacts of tracking devices on the species. The main aim of this study was to assess the impact of GPS-collars, including fitting the collar and wearing it, on the behaviour of the red panda. Seeing their endangered status and difficulty making observations in the wild, we opted to study the behaviour of captive red pandas. We studied the behaviour of two red pandas, a male and a female, in Rotterdam Zoo in The Netherlands before and after fitting GPS collars. Although we realize that no firm conclusions can be drawn from a study with only two individuals, we do believe the information provided by this study can only benefit the species and studies applying GPS collars in general.

## Materials and methods

### Study site and species

**Fig 1:**
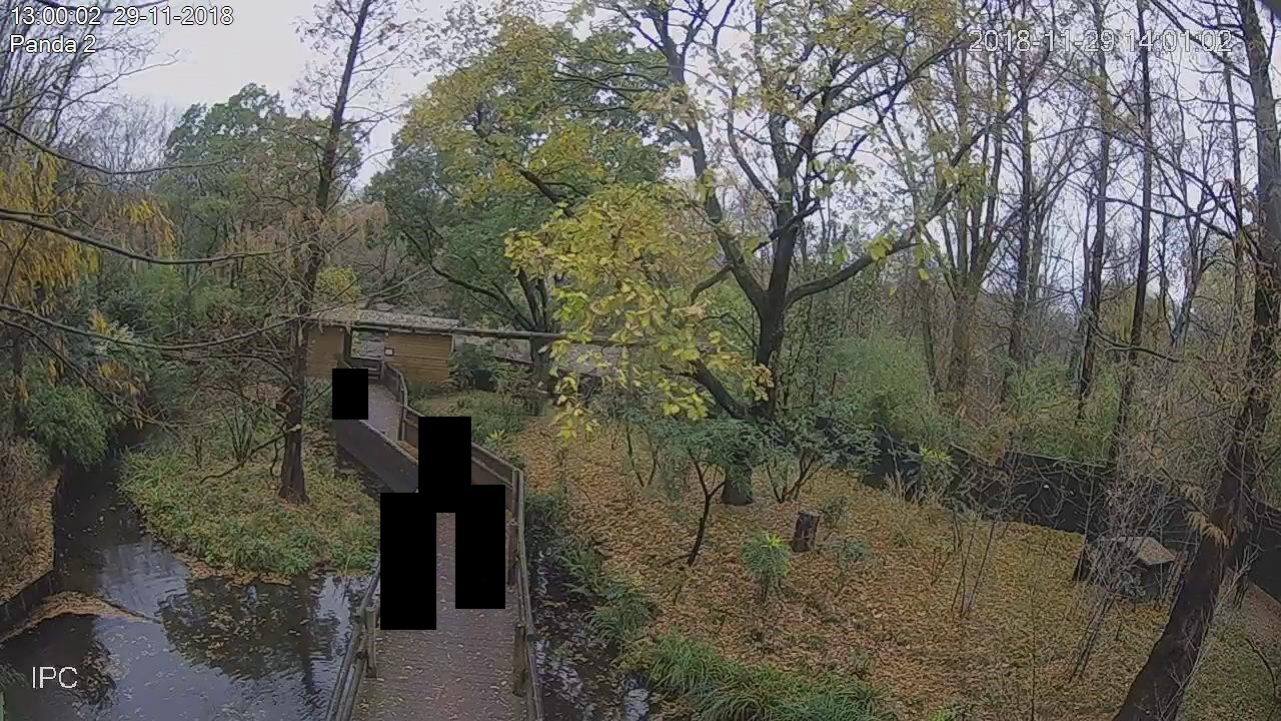
An overview of the red panda enclosure during the observation period. The indoor exhibit cannot be seen, but is situated in the below-right corner. A red panda can be seen in the tree on the left. Black squares are placed upon people that may otherwise have been recognizable.

**Fig 2:**
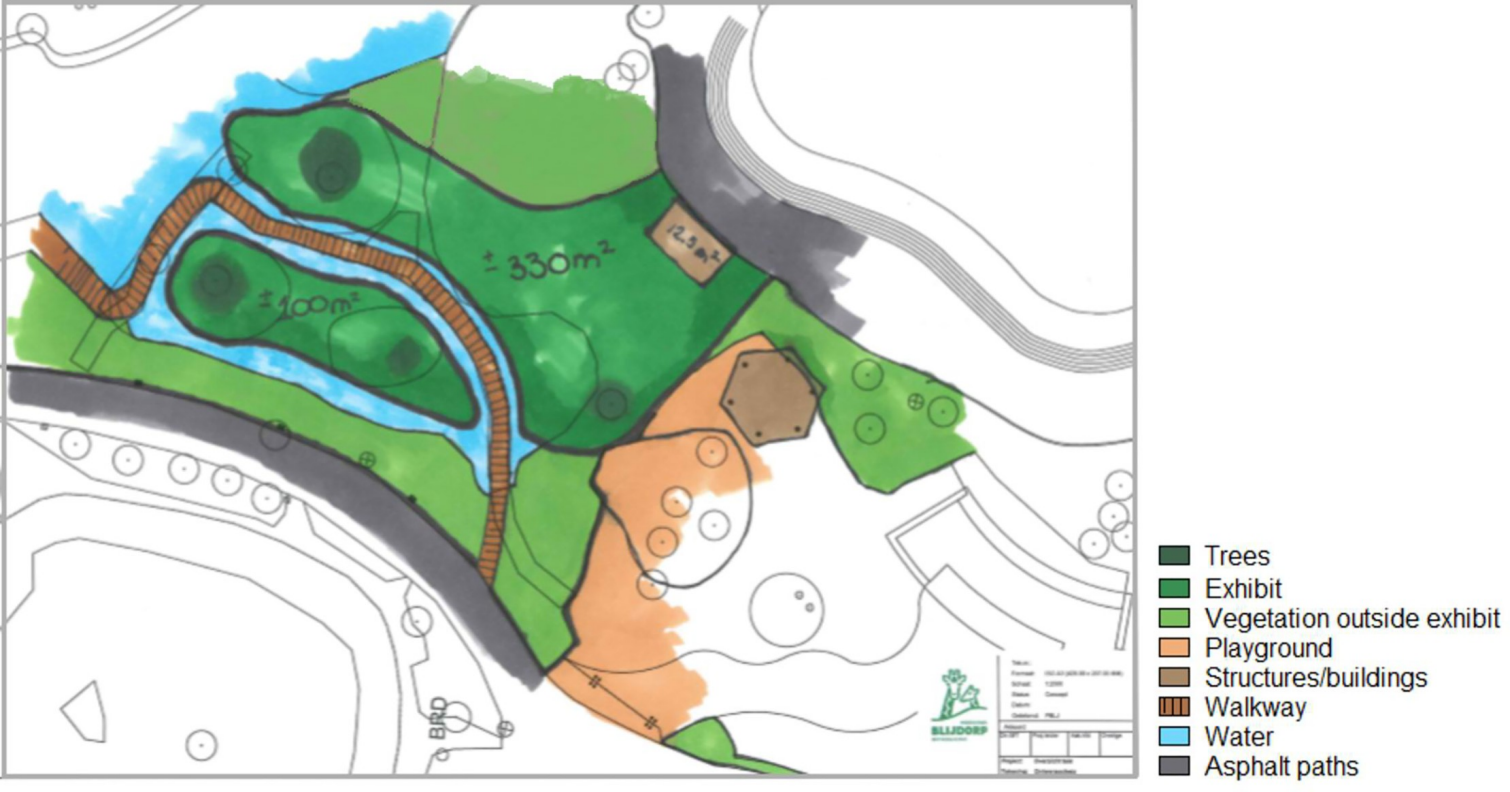
A ground plan depicting the red panda enclosure in Rotterdam Zoo. The 330m^2^ part is shared with the Michie’s Tufted Deer.

This study was carried out at Diergaarde Blijdorp, Rotterdam Zoo, Rotterdam, The Netherlands. Diergaarde Blijdorp is a modern zoo with a special focus on both *in-situ* and *ex-situ* red panda conservation. There is close cooperation with Red Panda Network (https://www.redpandanetwork.org/). It is an example of an IUCN ONE-PLAN approach where *in-situ* and *ex-situ* conservation complement each other [16]. Since 1978, a global red panda studbook has been kept by Rotterdam Zoo [17], which led to a healthy and diverse zoo population. The red panda enclosure is a naturalistic enclosure, measuring roughly 430 m^2^ (Figs 1 and 2). It houses seven large trees to climb and has three nest boxes and an indoor enclosure that is accessible day and night. There are two adult captive bred red pandas (*Ailurus f*. *fulgens*) in the enclosure, a male (Arjun) and a female (Yukiko) both 6 years old at the time of the study. The red pandas were housed together with their offspring, a male, born on 23 June 2018 and deceased on 6 December 2018 because of malnutrition. A correlation between the collaring of the mother animal and the death of the cub was thought highly unlikely. A pair of Michie’s Tufted Deer (*Elaphodus cephalophus michianus*) had access to half of the enclosure. The red pandas were fed twice daily. The first feeding was provided in the indoor enclosure around 07:30h and consisted of high-fiber panda pellets (DK Leaf-eater small pellets). The second feeding was provided in the outdoor enclosure around 15:00h and consisted of fresh cut bamboo. The area was accessible to visitors via a boardwalk, from 09:00–18:00h during summertime and from 09:00–17:00h during wintertime.

### Observations

Both the male and the female were individually observed during four 30-minutes observation sessions per day. The eight observation times started at 09:30h, 10:00h, 11:30h, 12:00h, 13:30h, 14:00h, 15:30h and 16:00h, and were randomly allocated to the male and the female. Baseline observations (before collaring) were conducted on 12 days between October 10^th^ 2018 till November 2^nd^ 2018. We therefore spend 24 hours observing the male and 24 hours observing the female before they were collared. State behaviours, which are longer lasting behaviours like walking, sleeping, eating, were recorded every 30 seconds through instantaneous sampling during the observation periods. Event behaviours, which are short behaviours like yawning and scent marking, were recorded six times per observation period through occurrence sampling. After collaring, which took place on 7 November 2018, both individuals were observed for in total 40 hours on 20 (the female) respectively 21 (the male) days between November the 8^th^ and December the 12^th^ 2018, following the same scheme as before collaring for the state behaviours. Event behaviour observations were taken whenever an event behaviour was observed every observation period. The discrepancy in number of observation days between the male and the female was because the cub fell ill and was, together with the female, transferred to the indoor enclosure on 3 December 2018. All behavioural observations of the female were therefore ceased to minimise stress levels for her and the cub. Two days later, the cub was transferred to the veterinary clinic, where it unfortunately died a day later. The female was released back into the enclosure after the cub was taken to the veterinary clinic, after which the observations were continued.

The behaviours were defined following the ethogram provided by Jule [18]. Additional behaviours were defined based on literature and during the observations. The ethogram used for this study contained 108 behaviours, which were roughly classified as four ‘inactive’ and 104 ‘active’ behaviours (see S1 Ethogram for a list of behaviours and their definitions). The active behaviours were further divided in ten subclasses: locomotive (N = 11), non-locomotive (N = 13), vocalisation (N = 3), territorial (N = 12), social (N = 30), maternal behaviours (N = 6), non-conspecific animal interaction (N = 9), keeper interaction (N = 8), consumption (N = 5), and stereotypies (N = 7). No hard division was made between state and event behaviours. While some behaviours are truly state behaviours, and some are truly event behaviours, most behaviours can be either based on time spent performing the behaviour. For instance, scratching self can be both an event and state behaviour.

At the start of each observation, ambient temperature was noted, because temperature can have an influence on the behaviour [19–21]. For the data analyses, three extra meteorological variables were added; air pressure (in hPa), cloud cover (from 0 [0% cover] to 9 [100% cover]) and relative air humidity (%). These data were sourced from the closest weather station operated by the Royal Dutch Meteorological Institute [22]. Six and eight temperature measurements were missing during the un-collared period for respectively the male and the female, because these were not properly documented. Two extra variables were added for the female; presence of the cub and age of the cub. All observations were performed by one observer (Wiene van de Bunte).

### GPS-collars

**Fig 3:**
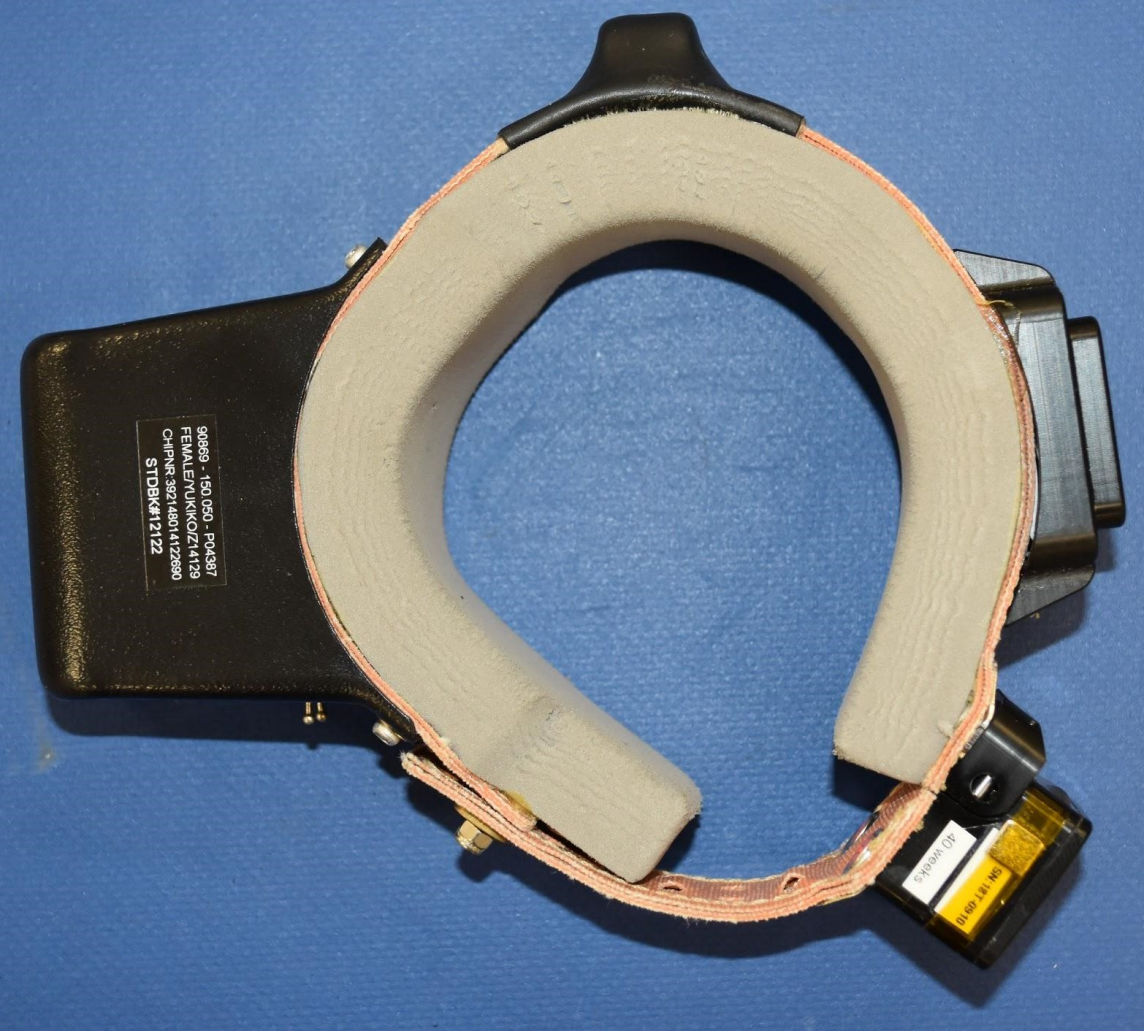
The GPS-collar used for the female red panda before deployment.

The red pandas were both collared on 7 November 2018 with a LiteTrack Iridium 150 GPS-collar. The dimensions of this tracking device were 43mm high x 74.92mm wide x 40.64mm deep. The devices, including collar, weighed 228 grams. This entailed no more than 5% of the body weight of the average adult red panda (5 kilogram), which falls within the 5–10% norm commonly used in ecological research [23]. The collar of the male had a 250 mm circumference and that of the female 220 mm. During the collaring it was found that the circumference for the male was on the large side. The collar had to be fixed on the tightest position, which left a relatively long loose part, which caused no problems during the study. When closed, the diameter of the females’ collar was 74.5mm and that of the male was 76.0mm. The grey foam (Fig 3) of the males’ collar seamlessly fitted around his neck, whereas the ends of the grey foam of the females’ collar did not join. There was a space of 2cm between the two ends of the grey foam. The animals were anaesthetized by the certified veterinarian of Rotterdam Zoo during a regular check-up for this procedure. Besides the collaring, the animals were checked by the veterinarian for any irregularities. During the collared period, the females’ neck was checked whenever she participated in training sessions. Training sessions depended on keeper availability and were done irregularly. The male did not partake in training sessions. On April 16th, 2019 (160 days after collaring) the females’ collar was removed. Two days later on April 18th, (162 days after collaring) the males’ collar was removed. As with the collaring, this was done during a veterinary check for which the animals were anesthetized.

### Data analyses

The counted event behaviours were transformed to mean frequency per observation. One new behaviour occurred after the collaring, this was ‘scratching collar’. This is defined as the regular scratching behaviour but directed to the collar and areas close around it. Since scratching the collar could only occur when the collar was present, the individual may have scratched itself instead. These two scratching behaviours were therefore combined in a separate ‘general scratching’ behaviour for the analyses. Data of the male and female were combined. Weather data variables were averaged between the observation sessions per day for the male and the female; e.g. the temperature during the first observation session of the male of day x was averaged with the temperature during the first observation session of the female of that same day. Data were analysed using R version 3.6.1 [24]. Because the data were not normally distributed, non-parametric statistical tests were used. Correlations were analysed using Spearman’s Rho, trend analysis was performed using linear regression. Group differences were analysed using either the Mann-Whitney U test or the Fisher’s exact test depending on the nature of the data. A number of behaviours were only displayed sporadically (Tables 1 and 2). Behaviours that were observed ten or fewer times were not taken into account for the statistical analyses.

**Table 1:** Frequency of observation and results of Wilcoxon test state behaviours.

State behaviours	N	N days	Both		Male		Female	
			W	p	W	p	W	p
General inactive classes	7638	32	1641	0.170	1477	**0.028**	1990	0.731
Cooling	6	1						
Laying- alert	2893	32	2270	0.086	2160	0.238	2166	0.216
Laying- sleeping	4364	32	1475	**0.028**	1351	**0.005**	1938	0.924
Out of sight	375	23	1771	0.372	1748	0.163	1885	0.803
General active classes	7722	32	2199	0.170	2363	**0.028**	1851	0.731
Climbing	973	32	1775	0.474	1772	0.436	1982	0.757
Fast climbing	5	3						
Jogging	15	11	1820	0.330	1896	0.448	1844	0.441
Subclass: locomotive	1399	32	1818	0.614	1766	0.418	1981	0.760
Out of sight active	35	6		** **			2111	**0.025**
Running	10	6	1917	0.977	1848	0.179	1991	0.305
Walking	361	32	1874	0.811	1819	0.413	1918	0.994
Subclass: non-locomotive	2515	32	2561	**0.002**	2399	**0.017**	2164	0.214
Defecating	17	15	1768	0.205	1768	0.092	1920	1.000
Grooming self	2178	32	2498	**0.004**	2373	**0.024**	2216	0.072
Scratching in general	27	10	1911	0.935	1888	0.601	1941	0.828
Sitting	213	31	2169	0.195	2083	0.186	2065	0.433
Standing	82	23	2111	0.253	1932	0.919	2110	0.208
Subclass: territorial	227	30	2442	**0.008**	2022	0.551	2416	**0.006**
Exploratory	131	29	1981	0.744	1879	0.773	1975	0.746
Vigilant re something inside enclosure	41	17	2359	**0.001**	2146	**0.047**	2237	**0.001**
Vigilant re something outside enclosure	53	19	2489	**<0.001**	2121	0.060	2363	**<0.001**
Subclass: social	46	16	2448	**<0.001**	2181	**0.014**	2291	**0.001**
Eye contact	1	1						
Playing	7	3						
Vigilant towards conspecific	38	14	2419	**<0.001**	2142	**0.031**	2271	**0.001**
Subclass: maternal behaviours	2531	27					1936	0.937
Allogrooming	19	8		** **			2133	**0.025**
Guiding cub	96	20					1711	0.167
In box	2344	26					1938	0.928
Maternal transport	72	11					1683	**0.037**
Animal interaction non conspecific	1	1						
Vigilant towards non conspecific	1	1						
Subclass: keeper Interaction	46	12	1964	0.693	2009	0.272	1878	0.604
Approach keeper friendly	5	1						
Vigilant towards keeper	41	12	1960	0.723	2008	0.274	1878	0.604
Subclass: consumption	957	30	1541	**0.045**	1732	0.218	1612	0.082
Drinking	40	20	1582	**0.021**	1819	0.207	1632	**0.034**
Eating browse	364	18	1699	0.116	1699	0.116	1668	0.069
Eating provision	1	1						
Food forage	89	15	1943	0.858	1835	0.345	2048	0.197

Significant differences are shown in bold.

**Table 2:** Frequency of observation and results of Wilcoxon test event behaviours.

Event behaviours	N	N days	Both		Male		Female	
			W	p	W	p	W	p
Approach keeper friendly	1	1						
Being touched	21	7					2259	**<0.001**
Cross bridge	47	16	1445	**0.001**	1927	0.924	1427	**<0.001**
Eye contact conspecific	36	16	1879	0.769	1848	0.179	1948	0.833
Eye contact non conspecific	4	4						
Lick cub	7	4						
Lick enclosure	8	7						
Pick up cub	18	11					1834	0.437
Scent mark	460	31	1747	0.380	1736	0.259	1888	0.866
Scratching in general	213	36	1603	0.107	1857	0.729	1670	0.140
Shake	214	21	571	**<0.001**	1128	**<0.001**	1084	**<0.001**
Sniff conspecific	2	2						
Sniff enclosure	190	32	1748	0.359	1892	0.860	1692	0.169
Sniff non conspecific	1	1						
Sniff young	16	7					1937	0.861
Stretching	31	15	2197	**0.041**	2103	0.065	2016	0.352
Take item	3	1						
Touch cub	127	21					1991	0.667
Try to pick up cub	191	20					1646	0.089
Vigilant re something inside enclosure	20	11	2226	**0.006**	2112	**0.025**	2033	0.131
Vigilant re something outside enclosure	63	24	2614	**<0.001**	2271	**0.014**	2283	**0.010**
Vigilant towards conspecific	56	21	1946	0.874	1929	0.948	1925	0.973
Vigilant towards keeper	13	10	1910	0.923	1927	0.924	1904	0.835
Vigilant towards non conspecific	10	9						
Vocalize twitter	166	21					1708	0.208
Watch cub	4	4						
Yawning	41	21	2078	0.290	2091	0.184	1954	0.744

Significant differences are shown in bold.

## Results

### Behavioural effects of the collar

The ambient temperature (Mann-Whitney U = 3197.5, *p* <0.001), air pressure (Mann-Whitney U = 2506, *p* = 0.004), and relative air humidity (Mann-Whitney U = 597, *p* <0.001) were all significantly different in the period before the red pandas wore the collar than in the period they wore the collar. During the period that the red pandas wore the collar it was colder (on average 8°C versus 15°C before) and the air pressure was lower (on average 1017hPa versus 1021hPa before), but the humidity was higher (on average 83% versus 70% before). The cloud cover was the same in both periods (Fisher’s exact *p* = 0.1986).

**Fig 4:**
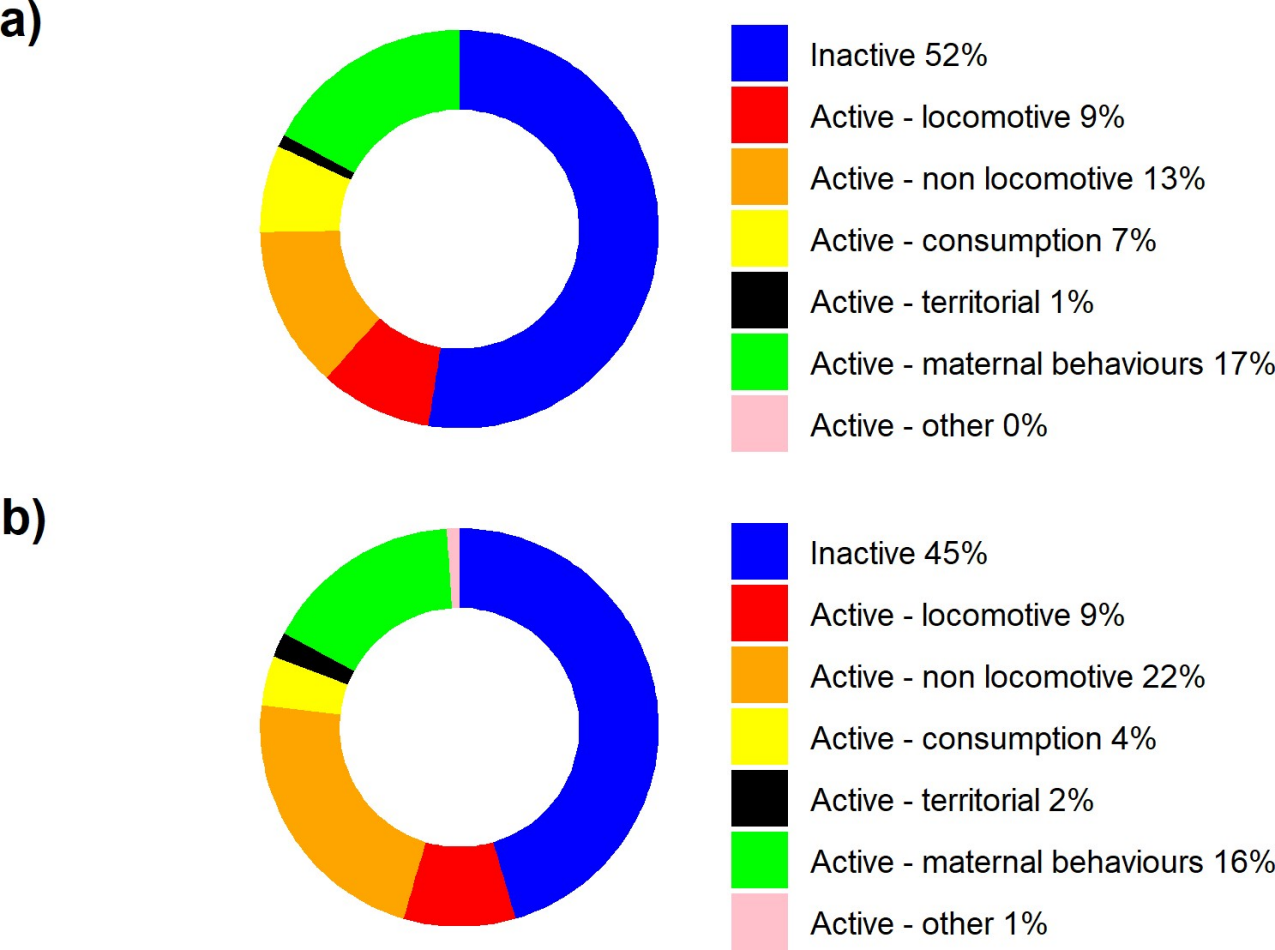
Main state behaviours (sub-classes) shown by the red pandas a) before collared, b) after being collared.

In total 2880 state behaviours were recorded during the un-collared period and 4800 during the collared period. The red pandas showed 53 of the 108 behaviours on the ethogram over the entire research period. The male showed 23 state and 17 event behaviours and the female showed 29 state and 28 event behaviours (Tables 1 and 2). Both the male and the female showed six behaviours that could be classified as both a state and an event behaviour (all five vigilance behaviours, eye contact, and approach keeper in a friendly manner, see Tables 1 and 2). Three state behaviours (fast climbing, eye contact, and approach keeper friendly) were only observed being performed by the male and nine only by the female (cooling, out of sight [active], playing, vigilant to non-conspecific, eating provision, and the four maternal behaviours; in box, maternal transport, allogrooming, and guiding cub). The 17 observed event behaviours performed by the male were also performed by the female. Before the red pandas were wearing their collar, 45% of the state behaviour observations were classified as inactive (Fig 4A). This percentage went up to 52% after collaring, mainly on the expense of being active but not locomotive (Fig 4B).

**Fig 5:**
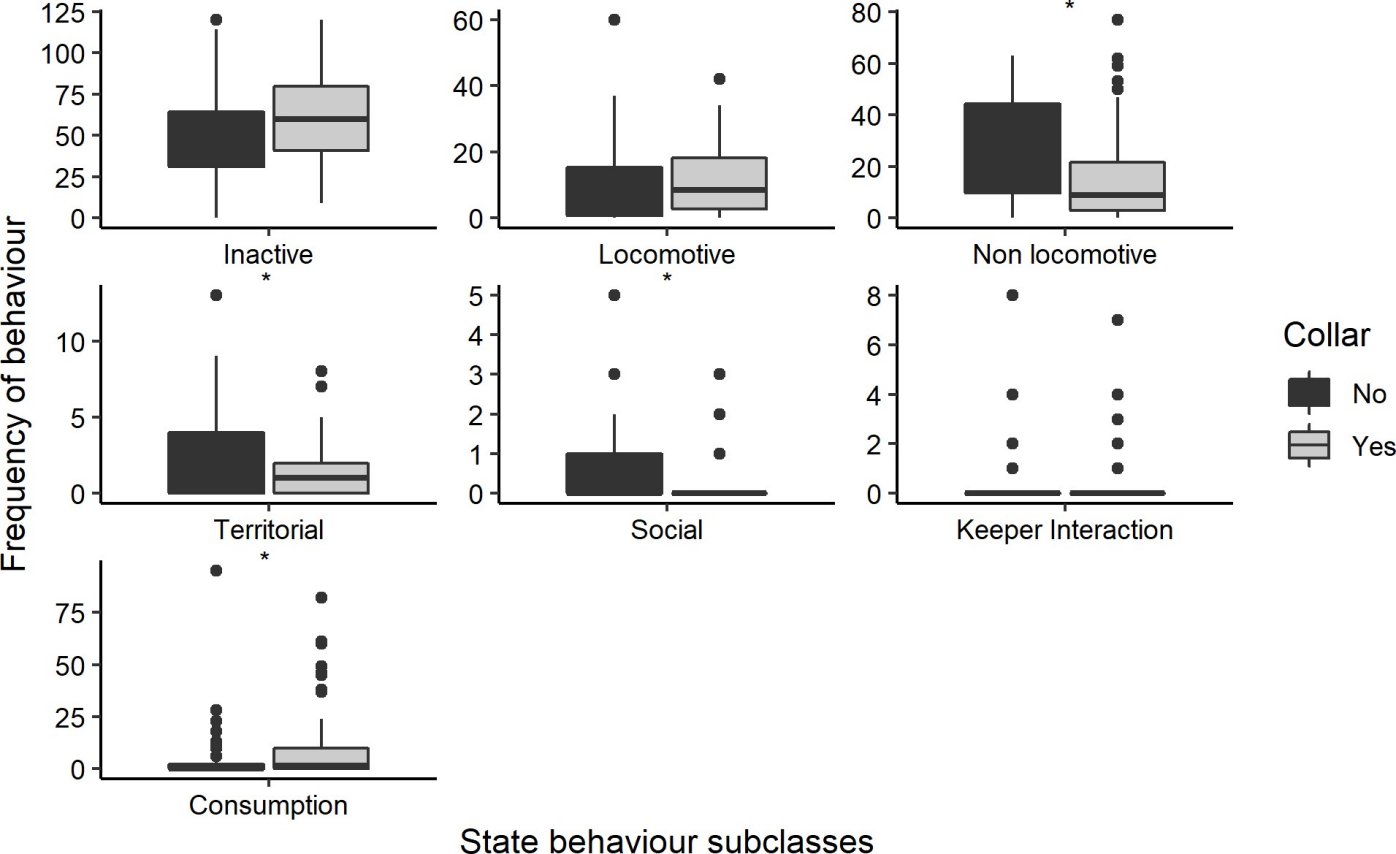
Differences between the frequency with which inactive and active state behaviours were displayed on a subclass level between the collared and uncollared period. Data for the male and female are pooled, only those behaviours that were displayed by both the male and the female are shown. * Indicates significant differences.

**Fig 6:**
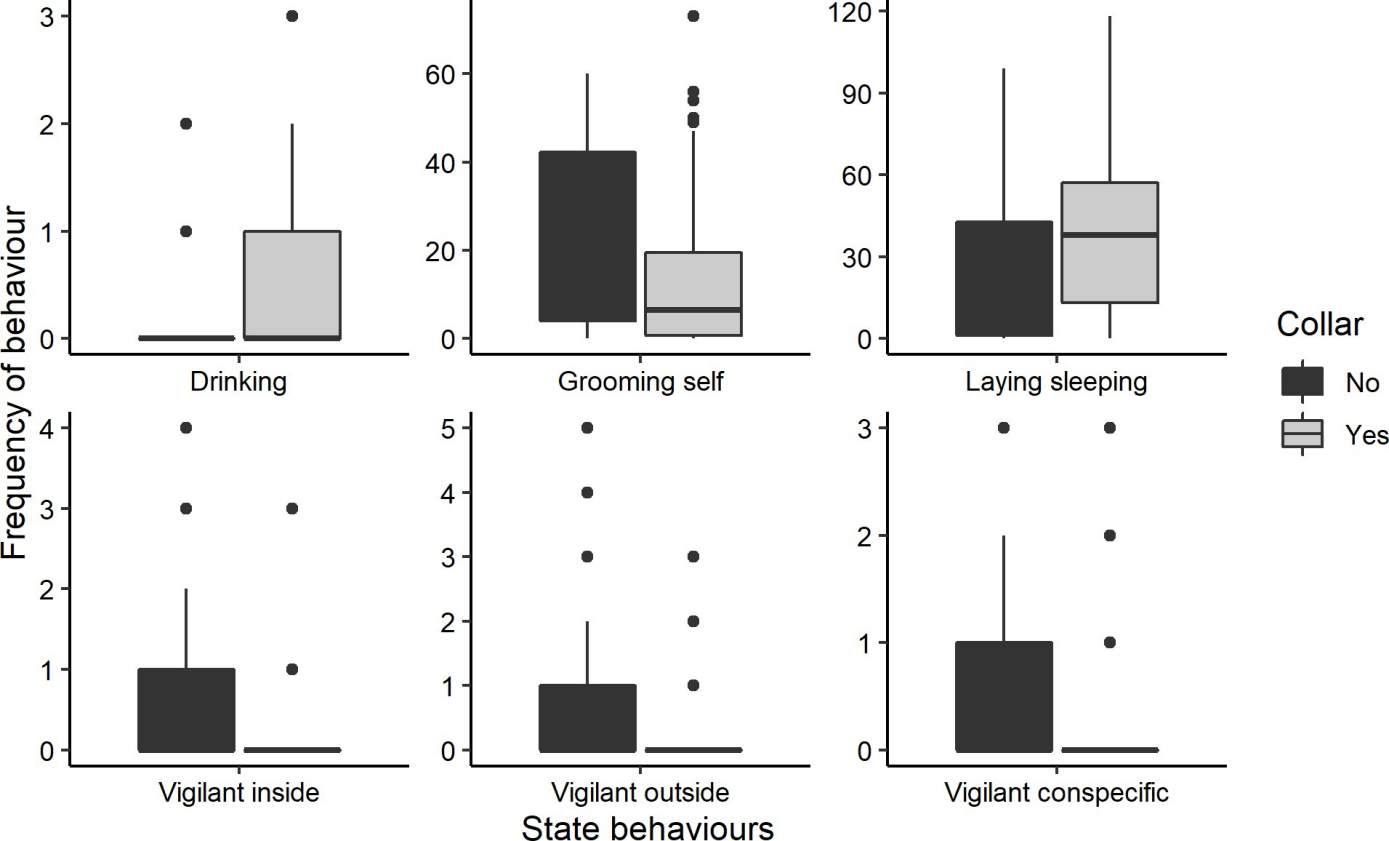
Significant differences between the frequency with which inactive and active state behaviours were displayed on a detailed level between the collared and uncollared period. Data for the male and female are pooled, only those behaviours that were displayed by both the male and the female are shown. Vigilant inside = vigilant towards something inside the enclosure, Vigilant outside = vigilant towards something outside the enclosure, Vigilant conspecific = vigilant toward conspecific.

Wearing the collar did not have significant impacts on the general time spend active versus inactive when data for the male and the female were pooled together (Table 1, Fig 5). However, whilst the collar did not have significant impacts on the amount of time the female was inactive, the male was significantly more often inactive during the un-collared than during the collared period, which was mainly due to a larger amount of time it spend laying sleeping (Table 1, Fig 6). When delving into the active behaviours of both individuals combined on a subclass level, significant differences in the frequency with which non-locomotive, territorial, social and consumption behaviours were displayed, were observed (Table 1, Fig 5). Consumption behaviours increased during the collared period and the other behaviours decreased. Whilst social behaviours differed significantly for both the male and female, non-locomotive behaviours differed significantly for the male but not for the female and territorial behaviours differed significantly for the female but not for the male. Consumption behaviours did not differ significantly for either the male or the female when tested separately.

When regarding the more detailed ethogram, there were several state behaviours that were significantly more or less commonly displayed after the red pandas were collared with a GPS collar (Table 1, Fig 6). When the behaviour of both the male and the female were pooled together the detailed state behaviours laying sleeping and drinking were displayed significantly more often when wearing the collar. Laying sleeping also significantly differed when only the data for the male was used and drinking when only the data for the female was used. The state behaviours vigilance towards something inside the enclosure (also significant for both the male and the female when regarded separately), outside the enclosure (also significant for the female), or towards a conspecific (also significant for both), and grooming self (also significant for the male) were displayed less often. A further three state behaviours were solely shown by the female and also significantly related to wearing the collar: being active but out of sight, maternal transport (both positively related), and allogrooming (negatively related). Of these nine state behaviours, the frequencies with which the three vigilance behaviours occurred were significantly positively correlated with the temperature (Pearson’s correlation Vigilance conspecific: t_120_ = 3.196, p < 0.001, Vigilance out: t_120_ = 2.598, p = 0.011, Vigilance in: t_120_ = 2.550, p = 0.012) and negatively with relative humidity (Pearson’s correlation Vigilance conspecific: t_126_ = -3.635, p < 0.001, Vigilance out: t_126_ = -2.649, p = 0.009, Vigilance in: t_126_ = -2.148, p = 0.034). The frequency of grooming self was significantly positively correlated with the air pressure (Pearson’s correlation t_126_ = 2.503, p = 0.014) and maternal transport negatively with the temperature (Pearson’s correlation t_120_ = -2.325, p = 0.022).

**Fig 7:**
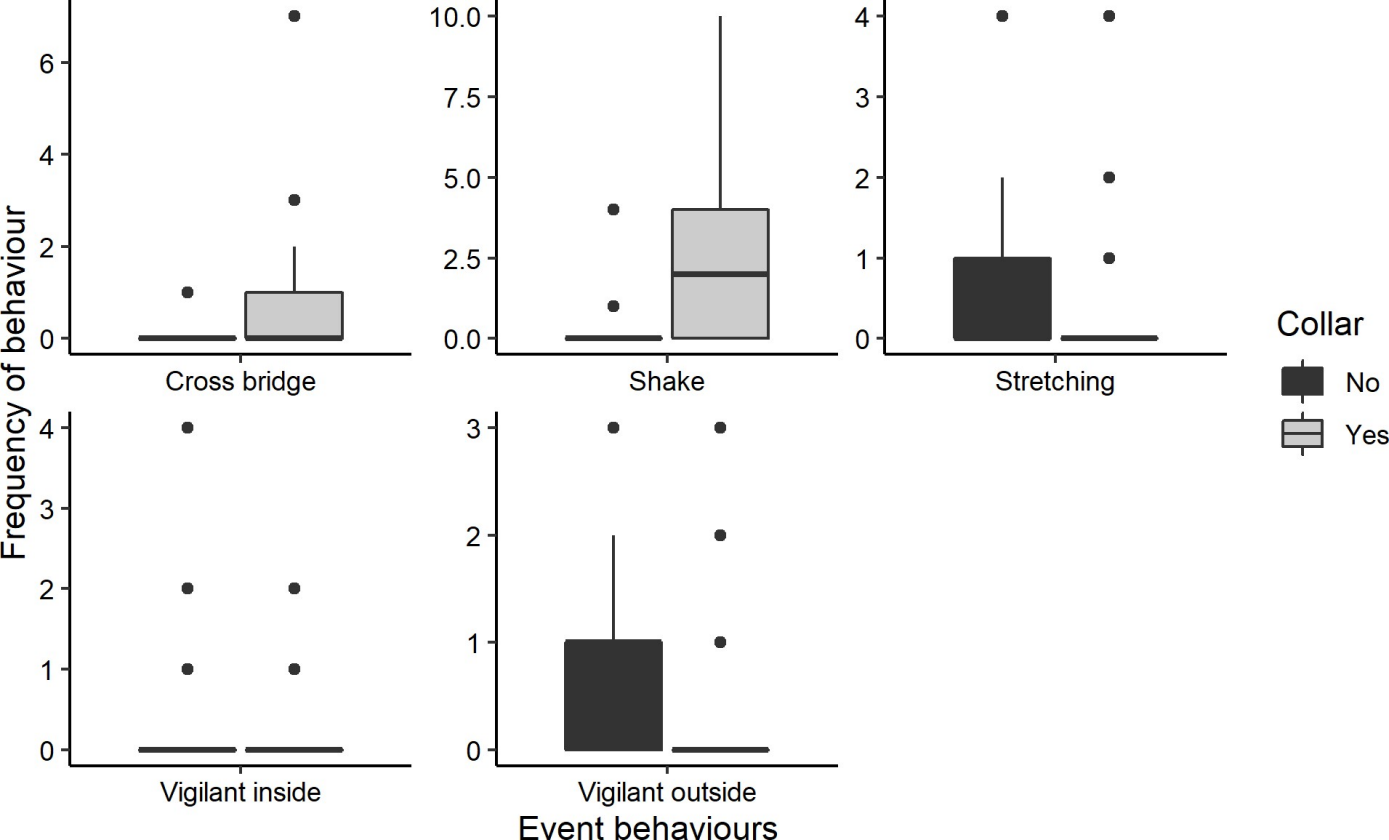
Significant differences between the frequency with which event behaviours were displayed between the collared and uncollared period. Data for the male and female are pooled, only those behaviours that were displayed by both the male and the female are shown. Vigilant inside = vigilant towards something inside the enclosure, Vigilant outside = vigilant towards something outside the enclosure.

Five event behaviours were significantly related to wearing the collar when data of both red pandas were pooled together (Table 2, Fig 7): vigilance towards something inside the enclosure (also significant for just the male), vigilance towards something outside the enclosure (also significant for both), shaking (also significant for both), crossing the bridge (not significant for either when data were not pooled) and stretching (not significant for either when data were not pooled). Shaking and crossing the bridge was more often observed after collaring, the other behaviours were more often displayed before collaring. The frequency with which shaking was displayed did not decrease over time (Adjusted R^2^ 0.004, *p* = 0.254). A further event behaviour, being touched, was significantly related to wearing the collar for the female (Table 2) and was significantly more often observed before wearing the collar. These six event behaviours were all significantly correlated with the temperature (Table 3). Being touched, crossing the bridge, shaking, and vigilance towards something inside the enclosure were also correlated with air humidity, and crossing the bridge with cloud cover (Table 3). Shaking and crossing the bridge were more often observed in colder temperatures and higher air humidity. Crossing the bridge was also observed more often in cloudy conditions.

**Table 3:** Results of the Pearson’s correlation test between weather variables and event behaviours that were significantly related to wearing the collar.

Weather variable	Event behaviour	t	df	p
Air humidity	Shake	4.6005	126	<0.001
	Cross bridge	3.6052	126	<0.001
	Being touched	-3.5566	126	0.001
	Vigilant re something inside enclosure	-3.5488	126	0.001
Cloud cover	Cross bridge	2.1845	126	0.031
Temperature	Shake	-4.0257	120	<0.001
	Cross bridge	-3.2629	120	0.001
	Being touched	2.62	120	0.010
	Vigilant re something outside enclosure	2.3606	120	0.020
	Stretching	2.1765	120	0.031
	Vigilant re something inside enclosure	1.9953	120	0.048

### Physical effects of the collar

During the check-ups at training sessions in which the female was tempted to voluntarily partake, no irregularities were found for the female. The last four weeks before the removal of the collar, training sessions were attempted, but the female did not want to participate. Therefore, the females’ neck was not checked during this period. Upon removing the collar, it was discovered that she had lost most of her hair under the collar and had developed several abrasions. The skin was subsequently cleaned and disinfected and treated with Dermiel skinspray (AstFarma) 1–3 times daily until full recovery. For the male only minor hair loss and no further skin damage was found.

## Discussion

### Behavioural effects of the collar

We have to acknowledge that we cannot draw strong conclusions from our study since 1) we only observed two red pandas, 2) we did not observe around the clock and only observed for a limited period of about four weeks, and 3) the observations were not done under a controlled set-up. We however did find several changes in the behaviour of the two individuals we observed after they were collared with a GPS collar. Several of these changes in their behaviour may be due to other factors not controlled for, such as factors related to the season, changes in the weather [20, 21], number and behaviour of visitors [25] or to the presence of the cub (such as the maternal behaviours) [26]. Other behavioural changes, such as the increase in frequency with which the bridge was crossed after collaring, may be just happenstance. Nevertheless, there were several significant behavioural differences that may directly be related to wearing a GPS-collar. Changes in the frequency with which the animals shake, scratch or groom themselves or other individuals may indicate direct effects of the collar. Scimitar-horned oryx (*Oryx dammah*) showed for instance short term increase of head shaking immediately after being fitted with GPS collars [27]. Also captive red foxes (*Vulpes vulpes*) showed significant increases in the behaviours rubbing and shaking after being fitted with telemetry collars. Like with the oryx, effects would diminish over the four week observation period, indicating a certain level of acclimatisation to the collars [28]. Global Navigation Satellite System collars had on the other hand little to no impact on the behaviour of cattle (*Bos taurus*) [29].

We did not find any significant relationships between the frequency with which the pandas scratched themselves (including the collar), but we did observe the shaking behaviour significantly more often after the animals were collared. There was no indication that shaking decreased again after some time, which may indicate a lack of acclimatisation to the collar. Our results further indicate that wearing a collar decreased the grooming behaviour. Decreased grooming may have negative physiological effects since grooming is frequently used to lower parasite (e.g. lice) loads [30, 31]. Also vigilance levels may be altered due to the presence of a collar, more attention being directed towards the collar than to the surroundings. In our study, three out of five vigilance behaviours observed were significantly less observed in the period the pandas were collared than before. It cannot be stated that this is caused by the collar alone, since several of these behaviours were also related to changes in the weather. The temperature during the un-collared period was for instance higher than during the collared period. There may be a combined effect of weather circumstances and the wearing of a collar that account for the observed differences. However, it may have severe repercussions for wild animals if GPS collars would indeed decrease vigilance levels, possibly leading to increased predation events. The multitude of studies investigating vigilance levels of wild animals using GPS collars may therefore benefit from pilot studies such as ours.

There have, to our knowledge, not been any previous ex situ studies on effects of GPS collars on the behaviour of red pandas, so comparing our results with that of other captive red pandas is thus far not possible. There have however been several in situ studies, but direct comparisons of their findings with ours are difficult due to different settings and methodology. Johnson et al. [12] for instance, used radio telemetry to monitor the movement, activity patterns and feeding behaviours of one female red panda in the Wolong reserve in China. They found that, although they only observed her on four occasions due to dense vegetation, she was crepuscular and on average active on 36.5% of the day while taking about two, four hour long resting periods per day. We did not observe the red pandas around the clock, so activity patterns cannot be compared directly. We however found that they were less active once collared (55% of active behaviours pre versus 48% after collaring). But, as already mentioned, this may have been due to other factors not related to the collar as well.

### Physical effects of the collar

With regards to physical responses to the collar; the female showed hair loss and skin abrasions which can have different causes. The diameter of the females’ collar was 74.5mm and that of the male was 76.0mm. The grey foam (Fig 3) of the males’ collar seamlessly fitted around his neck, whereas the ends of the grey foam of the females’ collar did not join. This resulted in a 2cm space between the ends of the grey foam of the females’ collar. This may have caused friction which caused the abrasions. It should be noted that during spring the red panda sheds its winter coat, which results in loose hairs. It is possible that these loose hairs got stuck between the neck and the collar of the female, causing more friction to occur.

### Drawbacks and recommendations

The emphasis here lies on an ideal study. In most zoo cases, ideal equals hypothetical. Due to the smaller population size and high costs, it is problematic to set up a study involving a representative sample size. In this study, the sample size was unfortunately only two. These two individuals were different in social setting, sex, and personality, based on reports from the keepers. The observations for both animals should thus ideally not be pooled, we therefore also presented results separately. However, several behavioural similarities with regards to the collar could be seen from both the male and the female, which gives some strength to our findings. To increase sample size, the experiment could have been conducted in different zoos. A recent trend in red panda management is the housing in single-sex groups. These groups are interesting candidates for a study like ours, since the individuals in these groups are comparable. However, the costs of doing a pilot study like ours are high, and if the results were to be negative, a lot of conservation money would be wasted. Another, often used technique, to increase *N* is the use of a surrogate species. The surrogate should be a comparable species, in ecology, habits and physiology. As stated, the red panda is a very unique animal in an ecological and evolutionary perspective [32]. This makes it nearly impossible to find a suitable surrogate. Even so, the red pandas in this study already are a surrogate for wild red pandas. The environment and interaction with people are vastly different for these zoo pandas, compared to their wild conspecifics. The pandas used in this study are both captive-born animals, which have known the zoo environment for their entire lives. It is therefore debatable how comparable these individuals are to wild individuals. The argument for a surrogate is applicable here too, the best surrogate is an animal that is highly comparable to the target species. In this case, the captive-bred red panda is the closest surrogate for the wild red panda.

Furthermore, we did not monitor the red pandas around the clock. Although a video set-up was in place, not the entire enclosure was monitored due to ethical reasons, and red pandas were often outside the view of the video camera. Furthermore, the observation period was limited to four weeks, which has shown to be enough to detect acclimatisation to a collar is other studies [28, 29], but may still not have been enough in our study since the increased shaking behaviour after collaring that was observed when the data for both red pandas were pooled together did not decrease over time. It is therefore advised that further studies observe for longer periods of time.

Another drawback of our study was that it was conducted in a non-controlled environment. As mentioned before, other effects like weather conditions [20, 21] as well as presence and behaviour of visitors [25] may have affected our study. To cope with possible confining environmental effects on our study, a change in study design is a solution. In an ideal situation, the observations are done in an area where the environment is completely controllable. However, such an environment may reflect natural situations less than the enclosure we used and behaviour of red pandas in the wild and in enclosures differ to some extent [18, 33, 34]. Results from such a study would therefore likely be less transferable to wild individuals than results from our study. Alternatively, a control group of individuals not wearing a collar could be observed simultaneously. We however did not have access to more red pandas that could be included as a control group in our study.

Even though the study environment we used was suboptimal and our sample size was low, we deemed this a workable setup for a pilot study. We however recommend further studies in a controlled set-up in other zoos or with wild individuals for a better understanding of the effects of GPS collars on red pandas. Ten adult wild red pandas in the temperate broad-leaf mixed and rhododendron forests of eastern Nepal have recently been fitted with GPS collars using our recommendations [35]. Results of that study will in due time most certainly add to our understanding of the effects GPS collars can have.

## Conclusion

This study contributes to current research by the finding that collaring can have both physical and potentially also behavioural effects on red pandas. The female showed hair loss and developed skin abrasions. This was likely caused by the non-optimal fit of the collar. Furthermore, several behaviours displayed by the female and, to a larger extent, by the male were altered after collaring. Although some of these behavioural changes may be happenstance or related to the weather or other factors; other behavioural changes, such as an increase in shaking and a decrease in grooming and vigilance, may be directly related to the collar. Since there was no strong behavioural effect of the collar found in this study, the collaring of wild red pandas, for means of data collection to promote the conservation of the species and its environment, is only recommended when an optimal fit of the collar can be assured. Based on our study, we recommend foam lining in the entire collar. Furthermore, behavioural observations of more (captive) individuals, under a controlled set-up, is recommended since we were only able to include two individuals under a non-controlled set-up in our study.

Besides the specific recommendations for the red panda, we stress that a pilot study under a controlled environment (zoo, rehabilitation centre) should be done before deploying collars in the wild with any species. By studying the behaviour of the captive animals, behavioural and welfare issues can be detected and possibly solved by a change in the study design. Besides the testing for effects on the animals, a general test of the proposed collars’ performance under different circumstances is recommended to understand the difficulties that influence the gathering of GPS data.

## Supporting information

S1 Ethogram Ethogram for the red panda, adapted from Jule (2008). (DOCX)

S1 Raw data All data collected is provided in the supporting information. (XLSX)

## References

[1] Cagnacci F, Boitani L, Powell RA, Boyce MS. Animal ecology meets GPS-based radiotelemetry: a perfect storm of opportunities and challenges. Philosophical Transactions of the Royal Society B: Biological Sciences. 2010; 365: 2157–2162. doi: 10.1098/rstb.2010.0107 20566493 PMC2894970

[2] Hebblewhite M, Haydon DT. Distinguishing technology from biology: a critical review of the use of GPS telemetry data in ecology. Philosophical Transactions of the Royal Society B: Biological Sciences. 2010; 365: 2303–2312. doi: 10.1098/rstb.2010.0087 20566506 PMC2894965

[3] Gursky S. Effects of radio transmitter weight on a small nocturnal primate. American Journal of Primatology. 1998; 46: 145–155. doi: 10.1002/(SICI)1098-2345(1998)46:2&lt;145::AID-AJP4&gt;3.0.CO;2-W 9773677

[4] Hamley JM, Falls JB. Reduced activity in transmitter-carrying voles. Canadian Journal of Zoology. 1975; 53: 1476–1478.

[5] Webster AB, Brooks RJ. Effects of radiotransmitters on the meadow vole, Microtus pennsylvanicus. Canadian Journal of Zoology. 1980; 58: 997–1001. doi: 10.1139/z80-139 7000327

[6] Brooks C, Bonyongo C, Harris S. Effects of global positioning system collar weight on zebra behavior and location error. The Journal of Wildlife Management. 2008; 72: 527–534.

[7] Matthews A, Ruykys L, Ellis B, FitzGibbon S, Lunney D, Crowther MS, et al. The success of GPS collar deployments on mammals in Australia. Australian Mammalogy. 2013; 35: 65–83.

[8] Krausman PR, Bleich VC, Cain JW, Stephenson TR, DeYoung DW, McGrath PW, et al. Neck lesions in ungulates from collars incorporating satellite technology. Wildlife Society Bulletin. 2004; 32: 987–992.

[9] Weber JM, Meia JS. The use of expandable radio collars for radio-tracking fox cubs. In: Priede IG, Swift SM, editors. Wildlife telemetry: remote monitoring and tracking of animals. New York: Ellis Horwood; 1992. pp. 698–700.

[10] Cypher BL. Effects of radiocollars on San Joaquin kit foxes. The Journal of wildlife management. 1997; 61: 1412–1423.

[11] Glatston A, Wei F, Than Zaw, Sherpa A. *Ailurus fulgens* (errata version published in 2017). The IUCN Red List of Threatened Species. 2015 e.T714A110023718 [Cited 24 February 2021]. Available from 10.2305/IUCN.UK.2015-4.RLTS.T714A45195924.en.

[12] Johnson KG, Schaller G., Jinchu H. Comparative behavior of red and giant Pandas in the Wolong Reserve, China. Journal of Mammalogy. 1988; 69: 552–564.

[13] Reid DG, Jinchu H, Yan H. Ecology of the red panda Ailurus fulgens in the Wolong Reserve, China. Journal of Zoology. 1991; 225: 347–364.

[14] Yonzon PB, Hunter ML. Ecological study of the red panda in the Nepal-Himalaya. In: Glatston AR, editor. Red panda Biology. The Hague: SPB Academic; 1989. pp. 1–7.

[15] Zhang Z, Hu J, Yang J, Li M, Wei F. Food habits and space-use of red pandas Ailurus fulgens in the Fengtongzhai Nature Reserve, China: food effects and behavioural responses. Acta Theriologica. 2009; 54: 225–234.

[16] Conservation Planning Specialist Group. The one plan approach to conservation. Conservation Planning Specialist Group. 2021 [Cited 2021 February 25]. Available from: http://www.cpsg.org/our-approach/one-plan-approach-conservation

[17] Glatston AR. The Red or Lesser Panda Studbook No. 1. Rotterdam: Stichting Koninklijke Rotterdamse Diergaarde; 1980. 7258039

[18] Jule KR. Effects of Captivity and Implications for Ex-situ Conservation: with special reference to red panda (*Ailurus fulgens*). Doctoral dissertation, The University of Exeter. 2008. Available from: https://ore.exeter.ac.uk/repository/handle/10036/65554

[19] McNab BK. Energy expenditure in the red panda. In: Glatston AR, editor. Red panda Biology. The Hague: SPB Academic; 1989. pp.73–78

[20] Fornelino MM, Giménez OS, Fidalgo de las Heras AM. (1995). Climatic influence on red pandas activity in Madrid Zoo. In: Glatston AR, editor. The Red or Lesser Panda Studbook No. 9. Rotterdam: Stichting Koninklijke Rotterdamse Diergaarde; 1995. pp. 31–39.

[21] Merchán M, Blasco T, Fidalgo AM, Sanz O. Climatic influence on red pandas (Ailurus fulgens) behaviour at Jerez zoo, Cadiz (Spain). In: Glatston AR, editor. The Red or Lesser Panda Studbook No. 10. Rotterdam: Stichting Koninklijke Rotterdamse Diergaarde; 1998. pp. 30–34. doi: 10.1523/JNEUROSCI.18-24-10603.1998

[22] KNMI, Klimatologi, Robert Leander. KNMI–Uurgegevens van het weer in Nederland; 2019 [cited 23 March 2019]. Database: [Internet]. Available from: http://projects.knmi.nl/klimatologie/uurgegevens/selectie.cgi

[23] Sikes RS, Animal Care and Use Committee of the American Society of Mammalogists. 2016 Guidelines of the American Society of Mammalogists for the use of wild mammals in research and education:. Journal of Mammalogy. 2016; 97: 663–688. doi: 10.1093/jmammal/gyw078 29692469 PMC5909806

[24] The R Foundation for Statistical Computing 2019. R version 3.6.1 (2019-07-05) — "Action of the Toes" Platform: x86_64-w64-mingw32/x64 (64-bit)

[25] Calero G, Fidalgo AM, Merchán M. The influence of visitors on the behaviour of red pandas Ailurus fulgens in Madrid Zoo. In: Glatston AR, editor. The Red or Lesser Panda Studbook No. 10. Rotterdam: Stichting Koninklijke Rotterdamse Diergaarde; 1998. pp. 14–17.

[26] Gebauer Alex. The Early Days: Maternal Behaviour and Infant Development. In: Glatston AR, editor. Red panda: Biology and conservation of the first panda. Norwich: William Andrew Publishing; 2011. pp. 157–191.

[27] Stabach JA, Cunningham SA, Connette G, Mota JL, Reed D, Byron M, et al. Short-term effects of GPS collars on the activity, behavior, and adrenal response of scimitar-horned oryx (*Oryx dammah*). PloS One. 2020; 15: e0221843. doi: 10.1371/journal.pone.0221843 32045413 PMC7012457

[28] Bruholt S. Behavioural responses of captive red fox (Vulpes vulpes) to telemetry collars. M. Sc. Thesis, Norwegian University of Life Sciences. 2018. Available from: https://nmbu.brage.unit.no/nmbu-xmlui/bitstream/handle/11250/2570999/Bruholt_2018.pdf?sequence=1&isAllowed=y

[29] Manning JK, Cronin GM, González LA, Hall EJ, Merchant A, Ingram LJ. The effects of global navigation satellite system (GNSS) collars on cattle (Bos taurus) behaviour. Applied Animal Behaviour Science. 2017; 187: 54–59.

[30] Tanaka I, Takefushi H. Elimination of external parasites (lice) is the primary function of grooming in free-ranging Japanese macaques. Anthropological Science. 1993; 101: 187–193.

[31] Hart BL, Hart LA. How mammals stay healthy in nature: the evolution of behaviours to avoid parasites and pathogens. Philosophical Transactions of the Royal Society B: Biological Sciences. 2018; 373: 20170205. doi: 10.1098/rstb.2017.0205 29866918 PMC6000140

[32] Salesa MJ, Peigné S, Antón M, Morales J. Evolution of the family Ailuridae: origins and Old-World fossil record. In: Glatston AR, editor. Red panda: Biology and conservation of the first panda. Norwich: William Andrew Publishing; 2011. pp. 27–41.

[33] Keller R. The social behaviour of captive lesser Panda (Ailurus fulgens) with some management suggestions. In: Glatston AR, editor. The Red or Lesser Panda Studbook No. 1. Rotterdam: Stichting Koninklijke Rotterdamse Diergaarde; 1980. pp. 39–56.

[34] Stevenson M, Anness L, Hanning J, Smith N. Red pandas at Edinburgh Zoo. In: Glatston AR, editor. Red panda Biology. The Hague: SPB Academic; 1989. pp. 103–114.

[35] Bista D, Lama ST, Weerman J, Sherpa AP, Pandey P, Thapa MK, et al. Improved trapping and handling of an arboreal, montane mammal: red panda *Ailurus fulgens*. Animals. 2021; 11: 921. doi: 10.3390/ani11040921 33805041 PMC8064068

