# Supplementary material for: Potential effects of GPS collars on the behaviour of two red pandas (Ailurus fulgens) in Rotterdam Zoo
Source: PLoS One. 2021 Jun 4;16(6):e0252456. doi: 10.1371/journal.pone.0252456 (PMC8177435; doi:10.1371/journal.pone.0252456)
Supplement: S1 Ethogram — (DOCX) [file pone.0252456.s001.docx]

## S1 Ethogram. Ethogram for the red panda, adapted from Jule (2008).

| **Behaviour** | **Description** |
| --- | --- |
| **Inactive** |  |
| Lying- alert | Head up, eyes open, reaction to surroundings in some manner (head or ear movement) |
| Lying- sleeping | Lying sleeping (either curled in ball or lying flat out)- unresponsive to noise/activity |
| Cooling | Lying flat out, limbs spread- only done in moderate up to very warm temperatures |
| Out of sight | Continuous stretch of time out of sight (believed to be inactive) |
|  |  |
| **Active** |  |
| *Locomotive* |  |
| Walking | Using all four limbs walking on ground, slow speed |
| Jogging | Using all four limbs jogging on ground, intermediate speed |
| Running | Using all four limbs running or bounding on ground, higher speed |
| Climbing | Moving along vertical or horizontal plane provided it is off the ground and not wider than one metre |
| Fast climbing | Running or bounding on non-horizontal plane or off ground, but no wider than one metre |
| Self play | Purposeless activity with self (i.e. rolling, tail chasing), but not repetitive |
| Out of sight | Briefly out of sight while moving |
| Hunt/stalk | Hunting/stalking of bird or other animal |
| Carry object | Carry object (e.g. bamboo, peacock feather) in mouth or hand while traveling (e.g. walking or climbing) |
| Out of sight | Believed to be active, but out of sight |
| Cross bridge | Climbing across the provided tree branch to move to another enclosure |
|  |  |
| *Non-locomotive* |  |
| Standing | Standing on all fours |
| Sitting | Sitting with front paws on the ground |
| Sitting- paws up | Sitting with front paws off the ground |
| Standing | Standing upright on two legs |
| Yawning |  |
| Shaking | Shaking the entire body whilst standing or sitting still |
| Stretching | Stretching the body and legs while standing |
| Scratching self | Using the legs to scratch the body |
| Scratching collar | Using the legs to scratch the collar and/or the areas adjacent to the collar |
| Grooming self | Cleaning the fur by using the tongue and/or paws |
| Hanging | Hanging from tree or enclosure furnishing |
| Defecating  Urinating |  |
|  |  |
| *Vocalisation (usually to con-specific or keeper)* |  |
| Quack-snort | Harsh, broad-band, polysyllabic |
| Twitter | Vocalisation between mother and cub. Resembles birdsong |
| Grunt | Short, deep |
|  |  |
| *Territorial* |  |
| Vigilance- in | Observation within enclosure (of a non con-specific) |
| Vigilance- out | Observation outside enclosure |
| Exploratory | Exploratory/territorial investigation of enclosure, can involve sniffing, interaction with furnishings within enclosure |
| Digging | Digging with front paws |
| Scenting | Rubbing of genital regions either sideways or front to back |
| Scratching | Using claws to rake across ground or object |
| Rubbing- muzzle | Rubbing of muzzle on ground or object |
| Rubbing | Rubbing of dorsal/lateral sides on ground or object |
| Sniffing | Olfactory investigation of an object or a non-animal |
| Licking | Olfactory investigation of an object or a non-animal |
| Tactile | Tactile investigation using paws to manipulate item |
| Digging | Extensive digging in ground, can include ‘rooting’ with muzzle in loose soil |
|  |  |
| *Social* |  |
| Eye contact | Two individuals making eye contact (stare) |
| Vigilance-con | One individual watching another (con-specific vigilance) |
| Physical avoid | Physical avoidance from a “reasonable” distance away |
| Displace- init. | Initiate physical displacement behaviour |
| Displaced- recip. | Recipient of displacement behaviour |
| Displacement-w | Displacement of another with no contact –Win |
| Displacement-l | Displaced by another with no contact – Lose |
| Initiate fight | Initiate physical aggression |
| Recipient fight | Recipient of physical aggression |
| Phys. fight-w | Winner of physical fight |
| Physical fight-l | Loser of physical fight |
| Chase | Chasing a con-specific |
| Chased Being | Chased by a con-specific |
| Grooming other | Initiate grooming session |
| Mutual groom | Mutual grooming session |
| Being groomed | Recipient of groom |
| Mutual touching | Close proximity or touching (while awake or sleeping) |
| Touching | Touching another con-specific |
| Being touched | Being touched by another con-specific |
| Sniffing other | Sniffing another con-specific, note* olfactory examination is amongst the most common type of social behaviour |
| Being sniffed | Being sniffed by another con-specific |
| Paws up | Standing up on hind paws- initiate |
| Playing | Mutual playing. May include rolling, non-agressive fighting, chasing |
| Following | Following a con-specific. Differs from chasing by walking speed |
| Being followed | Being followed by a con-specific. Differs from being chased by walking speed |
| Tail arching | Female arches tail, allowing for male inspection |
| Lordosis | Female takes a receptive position, inviting male to mount her |
| Mounting | Male mounts female |
| Copulation attempt | Copulation less than 30 seconds |
| Copulation | Copulation for longer than 30 seconds |
|  |  |
| *Maternal behaviours* |  |
| In box | In a nestbox, together with the cub |
| Allogrooming | Mother grooming the cub |
| Maternal transport | State of transporting the cub in the mouth |
| Pick-up cub | Event - trying to pick up the cub, results in failing or maternal transport |
| Guiding cub | Mother is pushing and guiding the cub to take it along with her |
| Touch | Touching cub friendly/voluntarily |
|  |  |
| *Animal Interaction (not conspecific)* |  |
| Vigilant | Vigilance/observation of animal |
| Eye contact |  |
| Approach-f | Approach animal- friendly |
| Approach-a | Approach animal- aggressive |
| Touched | Allow being touched by animal |
| Touch-f | Touching animal friendly/voluntarily |
| Touch-a | Touching (biting/scratching) animal aggressively |
| Sniffing | Sniffing another animal |
| Being sniffed | Being sniffed by another animal |
|  |  |
| *Keeper Interaction* |  |
| Vigilant | Vigilance/observation of keeper |
| Approach-f | Approach keeper- friendly |
| Approach-a | Approach keeper- aggressive |
| Take item (food) | Take an item from keeper (most likely food) |
| Touched | Allow being touched by keeper |
| Touch-f | Touching keeper friendly/voluntarily |
| Touch-a | Touching (biting/scratching) keeper aggressively |
| Climb | Climbing on keeper (friendly)- (not personally observed, but described) |
|  |  |
| *Consumption* |  |
| Drinking |  |
| Eating browse | Eating provisioned bamboo or browse in enclosure |
| Eating provision | Provisioned food- fruits, vegetables, pellets |
| Food forage | Foraging in enclosure (e.g. permanent trees, grass), can include digging |
| Digging | Digging with front paws |
|  |  |
| *Stereotypies* |  |
| stereotypy-1 | Purposeless locomotion (including walking and climbing), mostly repetitive, throughout the enclosure often in a figure-8 style, although route can vary to some extent |
| stereotypy-2a | Repetitive in a localized area- facing out towards public |
| stereotypy-2b | Repetitive in a localized area- facing in towards enclosure |
| stereotypy-3 circle | Repetitive walking/running in a tight circle, can be done on its own or within a pacing/stereotypic routine (Event behaviour) |
| stereotypy4 | Excessive mouth movements, i.e. tongue flicking |
| stereotypy5 | Excessive grooming/licking |
| stereotypy6 | Repetitive route in enclosure- predictable pattern, limited response/awareness to outside stimuli. In this case, accompanied by scent marking at repetitive locations but with no investigation (e.g. sniffing) |
